# The impact of *Filifactor alocis* on the severity of periodontitis among diabetic and non-diabetic patients: a narrative review

**DOI:** 10.3389/fdmed.2024.1408839

**Published:** 2024-05-22

**Authors:** Shumani Charlotte Manenzhe, Sandra Koutras, Nompumelelo Benedicta Zwane, Aubrey Isaac Masilana, Sindisiwe Londiwe Shangase

**Affiliations:** ^1^ Department of Periodontics and Oral Medicine, University of Pretoria, Pretoria, South Africa; ^2^ Department of Oral Medicine and Periodontics, University of the Witwatersrand, Johannesburg, South Africa

**Keywords:** filifactor alocis, periodontitis, diabetes mellitus, periodontal pathogenesis, periodontal pathogens, periodontal pocket, dysbiosis, host modulation

## Abstract

The extensive studies on *Filifactor alocis (Fa)* show a positive association with periodontitis, demonstrating elevated *Fa* levels compared to traditional periodontal pathogens in severe disease. Periodontitis is a chronic multifactorial disease induced by a dysbiotic microbiota in a susceptible host whilst diabetes is an established risk factor for periodontitis. Diabetes has been shown to alter the subgingival microbiota into distinct microbial communities which favours the shift towards disease. It is these very distinct subgingival microbiota that are believed to contribute to the high prevalence and severity of periodontitis in diabetic patients. This dysbiotic microbiota constitute traditional periodontal pathogens which include among others the red complex triad (*Porphyromonas gingivalis, Treponema denticola, Tannerella forsythia*), *Aggregatibacter actinomycetemcomitans*, the orange complex (*Fusobacterium nucleatum, Prevotella intermedia* etc.) and other emerging pathogens such as *Fa* that were previously unrecognised as role players in the pathogenesis of periodontitis. *Fa* is an asaccharolytic anaerobic gram-positive rod (AAGPR) currently considered to be one of the potential drivers of periodontitis progression and worsening through its unique virulence characteristics. Various mechanisms through which *Fa* contributes to the pathogenesis and severity of periodontitis have been reported. The mechanisms involved in the bidirectional relationship between periodontitis and diabetes are continuously being explored in order to enhance individualised preventative and management approaches in affected patients. This review aims to report on this emerging periodontal pathogen and its capacity to influence dysbiosis within a complex subgingival microbial community; including its potential role in the bidirectional relationship between diabetes and periodontitis. This review will highlight *Fa* as a potential prognostic indicator for disease worsening, which will help improve management protocols for periodontitis and diabetes.

## 1 Introduction

*Filifactor alocis* (*Fa*) is an asaccharolytic obligate anaerobic gram-positive rod (AAGPR), that was previously unrecognised and has made its way into the pathogenesis of the periodontitis arena. The pathogen is shown to be positively associated with periodontitis and endodontic infections (1). This marks a shift in the understanding of the bacterial aetiology in periodontitis, where the gram-positive organisms were believed to be associated principally with periodontal health whilst the gram-negative anaerobes were considered responsible for the initiation and progression of periodontal disease (2). This has led to exploration of the periodontal microbial community with identification of pathogens like *Fa* that were previously underappreciated and undetected in periodontitis. This could be attributed to the fact that *Fa* is a slow growing fastidious organism, and difficult to detect with conventional culture-based methods (3, 4). The 16S rRNA sequencing techniques and the targeted techniques such as checkerboard DNA-DNA hybridization and RNA–oligonucleotide quantification have enabled the identification of previously undetected species, in the aetiopathogenesis of periodontitis such as *Fa* (5).

Survival of *Fa* subgingivally, which is its main habitat, requires that the pathogen be able to establish itself, access nutritional support, evade host defence mechanisms, compete successfully with commensals and other periodontal pathogens to ensure its survival. This environment is altered in subjects with Diabetes Mellitus (DM), which impacts the pathogenicity of *Fa*. In a periodontal disease spectrum, *Fa* as a single pathogen has been isolated in high numbers in sites with gingivitis (6). On the other hand, when *Fa* establishes polymicrobial interactions/ consortia with other periodontal pathogens, its survival and virulence including that of other periodontal pathogens are enhanced; resulting in advanced periodontal destruction and severe periodontitis (6–9)*. Specific microbial consortia involving Fa: Fa + Aggregatibacter actinomycetemcomitans (Aa) + Dialister pneumosintes (Dp); Fa + Aa; Fa + Porphyromonas gingivalis (Pg) and Fa + Tannerella forsythia (Tf)* (3, 6, 10, 11) have been found to correlate with varied severity of periodontitis, thus supporting favourable interbacterial interaction. The establishment of polymicrobial synergistic interaction of *Fa* with these pathogens culminates in the release of proinflammatory cytokines thus enhancing *Fa's* invasive capacity (1). *Pg* has been shown to facilitate the uncontrolled growth of *Fa* in the same biofilm permitting the biofilm to become inflammophilic and thereby promoting periodontal tissue destruction (1). Furthermore, *Fa* and *Pg* may provide nutritional and adhesion support for each other (12).

The aim of this paper is to report on *Fa* as an emerging periodontal pathogen and its capacity to influence the pathogenesis of periodontitis in diabetic and non-diabetic patients. This review will also highlight *Fa* as a potential prognostic indicator for disease worsening, which will help improve management protocols for periodontitis and diabetes.

## 2 Association between diabetes mellitus and periodontitis

Diabetes mellitus (DM) and periodontitis have a bidirectional relationship that is well documented in many reviews and epidemiological studies. However, research to identify specific pathways linking these conditions is ongoing. The established relationship between DM and periodontitis is considered one of the significant determinants of the initiation and progression of periodontitis, with less predictability of the disease response to standard periodontal therapy (13). The co-existence of the two conditions leads to changes in the subgingival environment and the microbiome in periodontitis which favours the progression of periodontitis (14, 15). The risk for periodontitis is increased 2–3 times in people with uncontrolled DM proportional to the level of glycaemic control (16). Long-term studies have shown a higher occurrence of advanced periodontitis in patients with DM (17). In fact, periodontitis is considered the sixth complication of DM (14) after retinopathy, nephropathy, neuropathy, cardiovascular disease (17–20).

Diabetes Mellitus is a complex and common disease that includes several metabolic dysfunctions caused by a long-term state of hyperglycaemia, a hallmark of the disease. The hyperglycaemic status is generally as a result of decreased insulin secretion and action. Hyperglycaemia negatively impacts the host response by inhibiting the activity of neutrophils and increases the immune-inflammatory response which in the presence of periodontitis exacerbates periodontal tissue breakdown, whilst attempting to annihilate periodontal pathogens (3). This response results in the release of several pro-inflammatory mediators such as TNF-α, IL-1β and IL-6 (3), increased oxidative stress and disruption of the receptor activator of NF-*κ*B ligand/osteoprotegerin (RANKL/OPG) axis (21). All these factors result in worsening of periodontal tissue breakdown (20), which is the hallmark of periodontitis. Periodontitis is a chronic multifactorial inflammatory disease, induced by the polymicrobial dysbiosis state with resultant periodontal tissue destruction (22, 23). It is the sixth most prevalent chronic disease and a major health burden affecting over 750 million people worldwide (24).

The disruption of the host homeostasis caused by the pathogens from the red complex triad [*Pg*, *Treponema denticola (Td),* and *Tf];* orange complex *[Fusobacterium nucleatum (Fn), and Prevotella intermedia (Pi)];* and *Aa,* acting synergistically, is a driving factor for disease progression (23). Periodontitis similarly influences the progression, and management outcome of diabetes (25, 26). The periodontal pathogens and their by-products, together with inflammatory cytokines contribute to upregulated systemic inflammation. This leads to impaired insulin signalling and insulin resistance which worsens glycaemic control (20). Along with hyperglycaemia, the inflammatory response sustains the bidirectional relationship between DM and periodontitis (27). Inflammation, therefore, plays an important role in the severity of both conditions and sustains the negative effects that the two conditions have on each other.

## 3 The plausible mechanisms on how *Filifactor alocis* impacts on the severity of periodontitis

*Fa* exhibits a significant association with increasing periodontal probing depth and clinical attachment loss (3), hence rarely detected in healthy sites (7, 28). Schlafer et al. found an *Fa* prevalence of 77.8% and 76.7% amongst patients with generalized aggressive periodontitis (GAP) and chronic periodontitis (CP), respectively; whilst a low prevalence was observed among the periodontitis resistant group. This high prevalence in the two groups of patients translated to a higher occurrence of Fa in sites with deep periodontal probing depths (4–6 mm and 7–9 mm). Compared to the prevalence of the known putative periodontal pathogens, Fa was the third most prevalent amongst the GAP group, and the second most prevalent among the CP group; which places it at the same level as the other key players in periodontitis (29). In a different study, Kumar et al. found the incidence of *Fa* to be the highest; compared to the red complex triad, *Fn, Pi* and *Aa;* in sites showing worsening of periodontal health status (30). Even amongst patients with DM, the frequency of *Fa* is reported to be higher in those diagnosed with periodontitis when compared to those without periodontitis (3). Taken together, the data links *Fa* with severe forms of periodontitis as it shows a strong association with the clinical parameters indicating worsening periodontitis. Considering that DM, when poorly controlled, is associated with severe periodontitis, it is not farfetched to assume *Fa* involvement in these cases of severe periodontitis. This justifies *Fa* being considered a marker of periodontal tissue destruction and active disease (29, 31).

In the pathogenesis of periodontitis *Fa* is considered an inflammophillic pathobiont able to exploit the altered host environment to its advantage, taking over the disease process initiated by the so-called keystone pathogens like *Pg* and *Aa* and promote the progression of periodontitis (32). The co-occurrence relationships of certain bacterial networks in the various stages of the disease process, starting in health, gingivitis and periodontitis; highlights the role these bacteria play in disease progression. Of particular significance is the presence and interaction of *Fa* alongside the widely accepted periodontal pathogens in the gingivitis microbiome whilst also displaying a strong presence in the periodontitis microbiome; thus supporting its involvement in the disease progression model (33). For Fa to influence development and progression of periodontitis it should have the capability to survive the host environment and interaction with other periodontal pathogens. Not only is the survival of Fa facilitated through its ability to invade and establish itself within the periodontal pocket environment, in addition, its inflammophillic nature enables it to endure and take advantage of the inflammation (34).

The important determinants of *Fa* pathogenicity are its virulence attributes which facilitates its invasion of host cells, establishment, survival and persistence within the harsh subgingival environment of the periodontal pocket (1, 35). The putative virulence attributes of *Fa* which influence severity of periodontitis include: adhesion and invasion proteins; oxidative stress resistance; release of toxins that induce cell death; proteins that binds and inhibit complements particularly C3 which is key in complement activation cascade; manipulation or dysregulation of innate immune cells like neutrophils; and production of the adipokine visfatin.

### 3.1 Adhesion and invasion

Adhesion and invasion are a critical step for a pathogen to establish itself. *Fa* is able to adhere and invade epithelial cells, which is facilitated by the cell wall anchoring and other surface components whose expression is increased in the presence of *Pg* (1). When epithelial cells are coinfected with *Fa* and *Pg*, there is an increase in several membrane adhesion proteins and surface components which recognize adhesive matrix molecules which most likely mediate adherence and colonisation of host tissues during infection (12, 36). Thus, in the presence of *Pg*, the ability of *Fa* to invade epithelial cells is increased (31) leading to a much more pronounced immune response. The increased presence of *Pg* in diabetic patients further enhances the synergistic relationship which exists between *Fa* and *Pg* leading to a pronounced periodontal tissue destruction.

### 3.2 Oxidative stress resistance

One of the key mechanisms which is central to the survival and growth of *Fa* is its ability to resist and endure oxidative stress. This is unexpected given that *Fa* is an obligate anaerobe. Aruni et al. found *Fa* to be significantly more resistant to oxidative stress than other obligate anaerobes in the subgingival microbiome (7). This has been attributed to the sialidase activity, which apart from satisfying the *Fa* asaccharolytic needs through breakdown of sialylated glycoproteins in saliva; also results in the release of sialic acid by *Fa* that act as a reactive oxygen species (ROS) scavenger resulting in reduced oxidative stress within the periodontal pocket facilitating its survival (1). Hence *Fa*'s growth is favoured and sustained when placed under an oxidative stress environment of the periodontal pocket (37). The condition of increased oxidative stress is also noted in DM patients and is equally conducive for *Fa* growth and survival, as an environment that promotes the development and progression of periodontitis is created in this group of patients (3).

In addition, *Fa*'s ability to produce proteins that convert oxygen to more benign compounds also promote its growth and survival (38). One of these proteins is protein FA796, which is a superoxide reductase that converts superoxide radicals (produced by immune cells) into hydrogen peroxide (39). Hydrogen peroxide is less detrimental to cells than oxygen radicals such as superoxide, although it is still toxic (37). *Fa* has an innate ability to detoxify the local inflammatory periodontal pocket environment using the enzyme superoxide reductase (1). Another protein *Fa* possess is FA519, which plays a role in its survival and reproduction under hydrogen peroxide-induced stress, although the mechanism is unclear (37).

### 3.3 Proteases and toxins

Proteases have a vital role in virulence modulation among the major oral pathogens (40). *Fa* contains several proteases that have similar functional capacity as those found in the red complex triad (1). Amongst these, peptidases produced by *Fa* have been shown to bind and degrade type I collagen and gelatin via calcium-dependent route and also induce cell death of oral keratinocytes via caspase-independent apoptosis (8). *Fa* also contributes to periodontal tissue damage by inducing the release of neutrophil granule matrix content inclusive of matrix metalloproteinases (MMP) such as collagenase and gelatinase, with increased levels of MMP-1 observed in gingival tissue and crevicular fluid from patients with periodontitis (8, 41). As an asaccharolytic pathogen, *Fa* is able to thrive by degrading proteins to avail amino acids for its survival (1, 4, 42). The main amino acid substrate for *Fa* is arginine, whose degradation results in the production of butyrate (4). Butyrate is found in periodontal pockets, and is shown to affect the body's sensitivity to insulin (42). Hence when periodontitis and DM co-exist the levels of butyrate are increased and could play a role in aggravating the vicious cycle that exists between the two and exacerbating progression of periodontitis. Additionally, butyrate is reported to facilitate the recruitment of viruses to the periodontal microbial community, these viruses can be reactivated by butyric acid which is produced by *Pg* (43). The role and involvement of viruses in the pathogenesis of periodontitis has been elucidated. *Fa* may be contributing significantly to the presence of viruses (especially those from the human herpes family of viruses) found within periodontal pockets (44, 45). The production of butyrate is several times higher in certain periodontal pathogens like *Pg, Fn and Fa*; and contributes to the induction of oxidative stress and apoptosis of immune cells (4, 46) which may precede severe forms of periodontitis. Butyrate could be a better surrogate marker for a degree of inflammation and a predictor for severe periodontal diseases. Measuring butyrate could prove significant in cases where destruction has not occurred despite the presence of subclinical inflammation (46).

A novel RTX exotoxin FtxA believed to be contained within extracellular vesicles released by *Fa* has been identified (8, 11, 47). However, its role as a virulence factor contributing to the pathogenicity of *Fa* remains unclear (8). An analysis conducted by Bao and colleagues, revealed that a functional clustering of specific protein–protein interactions can discriminate between FtxA-producing and non-producing strains of *Fa.* However, further investigations are required to explore the identities, functions, and interactions of these proteinic groups to unveil whether they contain a pathogenicity island within *Fa* that could regulate the virulence of this species (48).

### 3.4 Host cell modulation

*Fa* is able to modulate host response by inducing the local production of proinflammatory cytokines including IL-1β, IL-6 and TNF-α within the periodontal tissues and also contributes to their systemic production (49). DM is shown to modulate the host response and bring about a hyperinflammatory state within the periodontium, which result in an increase of proinflammatory cytokines in relation to anti-inflammatory cytokines in shallow and deep sites (50). Moreover, Miranda and colleagues demonstrated an increased proinflammatory to anti-inflammatory cytokine ratio in patients diagnosed with DM and periodontitis (DMP) when compared to that seen in patients with periodontitis only (49). These in addition to *Fa* oxidative stress resistance and complement inhibition ability results in a prolonged inflammatory process that is detrimental to the host. Another way *Fa* modulates the host response is via the metabolites from degraded amino acids which result in an over activation of the immune response and further production of pro-inflammatory cytokines (51). Thus, the coexistence of DM and periodontitis could be expected to result in an even more increased expression and production of pro-inflammatory cytokines when compared to that seen in periodontitis alone (49). These proinflammatory cytokines may upregulate pathways promoting osteoclast generation and thus increase alveolar bone loss leading to disease progression (52). Through its various host modulation mechanisms, *Fa* aggravates the proinflammatory effects of DM on periodontitis and could thus contribute to severe tissue destruction seen in DMP.

Recent studies have shown a significant elevated level of COX2 expression by gingival fibroblast and monocytes in patients with periodontitis. COX2 plays a role in mediating periodontal tissue damage by converting arachidonic acid into prostaglandin E2, which in turn alters connective tissue metabolism and increases osteoclastic bone resorption (8).

### 3.5 The impact of *Filifactor alocis* on neutrophils

Neutrophils are the first line of innate immunity which are able to detect, localise and destroy the invading periodontal pathogens via phagocytosis, degranulation, production of reactive oxygen species and formation of neutrophil extracellular traps (NETs). *Fa* is able to interact with neutrophils through both direct contact and distal signalling, manipulating their effector functions and thus contributing to periodontal disease progression (9). In addition, *Fa* is able to direct migration of neutrophils via induction of IL-8 produced by epithelial cells which results in exocytosis of specific gelatinase granules and secretory vesicles, leading to a dysregulated and sustained inflammation and tissue damage (8, 43).

Although *Fa* is easily phagocytized, it remains viable within neutrophils for up to 4 h post-infection as it is able to evade intracellular neutrophil killing by preventing phagosome maturation, the release of lysozyme, lactoferrin, and other antimicrobial peptides against its phagosome (7, 38). These properties of *Fa* also hinders the neutrophil constitutive apoptotic program (9) which it exploits for its survival. *Fa* also significantly reduces the ability of neutrophils to produce NETs and intracellular ROS (38). This assists *Fa* to survive within the periodontal pockets and also assists in the survival of other members of the dysbiotic community (9). *Fa* survival within a pocket laden with neutrophils result in their accumulation in a dysfunctional state and release of mediators detrimental to the periodontal tissues resulting in abscesses or exudate. This could explain the presence of abscesses or exudate in severe forms of periodontitis and in DMP.

Diabetes mellitus also disrupts the host response resulting in compromised immunity involving various immune cells including neutrophils, macrophages and T-lymphocytes. The impaired immune response in DM makes diabetic patients prone to infection. Therefore, the effect of *Fa* on neutrophils combined with the immune dysfunction significantly contributes to the advancement and exacerbation of periodontitis observed in diabetic patients.

### 3.6 Inhibition of the host complement system

The complement system plays a significant role in the clearance and destruction of periodontal pathogens by phagocytes and cell lysis via formation of membrane attack complexes (11). *Fa* is able to evade the complement system via *Fa* complement inhibitor (FACIN), a novel cytoplasmic protein that can be secreted or expressed on the bacterial cell surface (53). This cytoplasmic protein FACIN has been classified as a rare potent complement inhibitor as it is able to bind to and inhibit C3 thus suppressing all three complement pathways (8, 11, 53). The effect of FACIN in the setting of periodontitis contributes to disease progression.

### 3.7 Production of visfatin

Another way *Fa* contributes to modulation of the host response favouring tissue destruction is through the adipokine, visfatin. This adipokine is increased in serum and gingival crevicular fluid of patients with periodontitis when compared to their non-periodontitis counterparts (54). *Fa* stimulates the synthesis and production of visfatin from macrophages found in gingival sites of patients with periodontitis in a dose dependent manner (55). Visfatin promotes the release of interleukin (IL)-1β, IL-6, tumour necrosis factor-α (TNF-α) and matrix metalloproteinases (MMPs) which leads to increased inflammatory processes responsible for severe periodontal tissue destruction (55–57). *P. gingivalis* and *F. nucleatum* also increase the synthesis and production of visfatin from gingival fibroblasts (57). These periodontal pathogens work synergistically with *Fa*, resulting in a local increase of visfatin within gingival tissues. Interestingly, the levels of Visfatin are also elevated in the serum of patients with obesity, diabetes mellitus, cardiovascular disease, and metabolic syndrome, conditions all closely associated with periodontitis (55–57). Thus, the local and systemic production of visfatin could represent one of the potential pathways through which the bidirectional relationship between microbial induced periodontitis and systemic conditions like DM is established.

## 4 *Filifactor alocis* in diabetic and non-diabetic patients with periodontitis

Although there are no phenotypic differences in clinical presentation of periodontitis in DM and non DM patients (15), the prevalence, extent and the severity of periodontitis is often increased in DM patients with multiple recurring periodontal abscesses (58). The microbial flora in periodontal pockets of DM subjects has been found to differ from that found in periodontal pockets of non-DM subjects (3, 50). Whilst a study by Duarte et al., reported low counts of *Fa* in DM vs. non-DM patients with periodontitis, other studies have reported significantly higher counts of *Fa* among DM patients as compared to non-DM patients with periodontitis (3, 31, 42, 59). From these studies we can conclude that although *Fa* is found in all periodontitis patients, its presence is increased amongst those with diabetes.

A recent study assessed the frequency of *Fa* in type 2 DM subjects with periodontitis vs. those who have a healthy periodontium and its correlation with clinical parameters. Outcomes of the study revealed a significantly greater number of subjects with *Fa* in the periodontitis group as compared to the healthy periodontium group (3). *Fa* is shown to dominate the subgingival microbiome of periodontitis patients with DM (31). These findings corroborate the study by Ganesan and colleagues who reported the dominance of *Fa* along with other periodontal pathogens in DM subjects when compared to non-DM subjects (59). The high levels of *Fa* in DM can be attributed to increased intracellular oxidative stress that develops as a consequence of microangiopathy mediated by hyperglycemia; and an exaggerated immune-inflammatory response to a dysbiotic microbiome (36, 59). Since *Fa* is reported to be resistant to oxidative stress, it is able to colonise and persist within the stressful periodontal pocket's environment where its growth is stimulated (3, 29). Consequently, the periodontal pocket environment in DM patients results in increased levels of *Fa* and supports its pathogenicity and facilitates the progression of periodontitis (3). Furthermore, a study by Kumar et al., found the incidence of *Fa* to be the highest; compared to the red complex triad*, Fn, Pi* and *Aa;* in sites showing worsening of periodontal health status (30). Thus, *Fa* exhibits a significant association with increasing periodontal probing depth and clinical attachment loss, which are significantly increased in the presence of *Fa* (3). Taken together, the data links *Fa* with severe forms of periodontitis as it shows a strong association with the clinical parameters indicating worsening periodontitis. In support of the association between *Fa* and worsening periodontitis, an intervention study conducted by Ashwini et al., showed notable decrease in *Pg* and *Fa* following non-surgical periodontal therapy and improved clinical periodontal parameters (31). This justifies *Fa* being considered as a marker of periodontal tissue destruction and active disease (29, 31).

## 5 The synergistic effects of *Filifactor alocis* and hyperglycemia

The periodontal pathogens and their by-products, together with inflammatory cytokines contribute to upregulated systemic inflammation. This leads to impaired insulin signalling and insulin resistance, thus resulting in hyperglycemia (20). Hyperglycemia compromises the host response by inhibiting the activity of neutrophils and increasing the immune-inflammatory response in an attempt to annihilate periodontal pathogens (3). This response overlaps with some of *Fa*'s virulence properties which result in the release of several pro-inflammatory mediators such as TNF-α, IL-1β and IL-6 (3), increased oxidative stress, and disruption of the receptor activator of NF-κB ligand/osteoprotegerin (RANKL/OPG) axis (21). All these factors in the presence of *Fa* can be expected to result in marked periodontal tissue destruction and/ or worsening of periodontitis. A study by Ganesan and colleagues demonstrated an association between distinct subgingival microbial clusters and hyperglycemia (59). They found an increased frequency of *Fa* with uncontrolled chronic hyperglycemia. These findings were similar to those made in a study that highlighted a significant negative correlation between glycemic control and *Fa* (3). The same authors also found a positive correlation between increased probing depth and clinical attachment loss in the presence of *Fa*. One can therefore extrapolate that increased *Fa* is correlated with hyperglycemia and worsening of periodontitis.

*Tf,* a periodontal pathogen that has been shown to have a synergistic relation with *Fa* is also reported to have a negative effect on glycemic control (3)*.* The co-occurrence of the two pathogens thus accelerate the worsening of periodontitis and poor glycemic control in patients with DM (3). The impact of *Fa* on glycemic control strengthens the bidirectional relationship between periodontitis and DM. Wherein DM increases the risk for periodontitis and/ or worsening thereof whilst periodontal inflammation induced by *Fa* has a negative impact on glycemic control. Reduction in *Fa* load is reported to favour improved glycemic control as shown in decreased HbA1c levels and increased insulin sensitivity (31). Moreover, individuals with good glycemic control show a significantly lower detection of *Fa* as compared to fair- and poor-glycemic-control subjects (3). Therefore, good glycemic control may lower the prevalence of *Fa* and lowering or inhibiting colonisation of periodontal pockets with FA can contribute to improved glycemic control. Along with hyperglycemia, inflammatory responses in periodontitis sustain the bidirectional relationship between DM and periodontitis (27). Considering that the main risk factor of periodontitis is DM, and the severity of periodontitis affects glycemic control, there is no doubt that *Fa* is an important pathogen to be studied in patients with diabetes. Paucity of studies concerning the role of *Fa* on glycemic control, is a limitation to conducting systematic reviews which could inform better approaches to glycemic control.

## 6 Conclusion

Notwithstanding, the impact of the historically well documented and common periodontal pathogens the red complex triad, orange complex species and *Aa*; the discovery of *Fa* and its role in the pathogenesis of periodontitis adds a new dimension to the progression and severity of periodontitis. This obligate anaerobe has a strong association with periodontitis, presenting with a higher prevalence in individuals with periodontitis. *Fa* has a significant impact on the pathogenesis of periodontitis through various mechanisms linked to its virulence attributes, in both diabetic and non-diabetic patients. Its synergistic relationship with putative periodontal pathogens and association with severe periodontitis, as marked by increased probing depths and increased clinical attachment loss makes *Fa* a key role player in the progression and severity of the disease. In patients with diabetes this impact is even more pronounced, justifying the severity of periodontitis that is generally seen in poorly controlled diabetic patients. This is further enhanced by the synergistic relationship that exists between *Fa* and hyperglycaemia worsening the disease state. The presence of *Fa* in diseased sites could therefore be a marker for an increased risk for having severe periodontal disease**.** Future well-designed longitudinal and molecular studies on Fa in diabetic and non-diabetic patients with periodontitis are needed to further understand this emerging periodontal pathogen and thus guide prognosis and management approaches. The knowledge gained from such studies will inform and expedite precise periodontal therapy focused on elimination of specific periodontal pathogens and host response modulation.
